# Antigenic and molecular characterization of low pathogenic avian influenza A(H9N2) viruses in sub-Saharan Africa from 2017 through 2019

**DOI:** 10.1080/22221751.2021.1908097

**Published:** 2021-03-23

**Authors:** Maxime Fusade-Boyer, Fidélia Djegui, Komla Batawui, Denis K. Byuragaba, Jeremy C. Jones, Fred Wabwire-Mangeni, Bernard Erima, Gladys Atim, Qouilazoni A. Ukuli, Titus Tugume, Koffi Dogno, Komlan Adjabli, Mvibudulu Nzuzi, Rachidatou Adjin, Trushar Jeevan, Adam Rubrum, Wolali Go-Maro, Ghazi Kayali, Pamela McKenzie, Richard J. Webby, Mariette F. Ducatez

**Affiliations:** a IHAP, UMR1225, Université de Toulouse, INRAe, ENVT, Toulouse, France; b LADISERO, Parakou, Benin; c Laboratoire Central Vétérinaire de Lomé, Lomé, Togo; d Makerere University, Kampala, Uganda; e Department of Infectious Diseases, St. Jude Children’s Research Hospital, Memphis, Tennessee, USA; f Makerere University Walter Reed Project, Kampala, Uganda; g Human Link, Hazmieh, Lebanon; h University of Texas Health Sciences Center, Houston, Texas, USA

**Keywords:** Influenza virus, Africa, phylogeny, antigenic cartography, one health

## Abstract

Sub-Saharan Africa was historically considered an animal influenza cold spot, with only sporadic highly pathogenic H5 outbreaks detected over the last 20 years. However, in 2017, low pathogenic avian influenza A(H9N2) viruses were detected in poultry in Sub-Saharan Africa. Molecular, phylogenetic, and antigenic characterization of isolates from Benin, Togo, and Uganda showed that they belonged to the G1 lineage. Isolates from Benin and Togo clustered with viruses previously described in Western Africa, whereas viruses from Uganda were genetically distant and clustered with viruses from the Middle East. Viruses from Benin exhibited decreased cross-reactivity with those from Togo and Uganda, suggesting antigenic drift associated with reduced replication in Calu-3 cells. The viruses exhibited mammalian adaptation markers similar to those of the human strain A/Senegal/0243/2019 (H9N2). Therefore, viral genetic and antigenic surveillance in Africa is of paramount importance to detect further evolution or emergence of new zoonotic strains.

## Introduction

The low pathogenicity avian influenza (LPAI) H9N2 virus is the most widespread subtype in poultry around the world, posing a concern for animal and public health [1,2]. In poultry, H9N2 infections generally lead to mild clinical signs, making its detection in the field difficult. However, severe morbidity can occur in cases of coinfection with bacteria or other viruses [3]. In humans, at least 59 cases have been reported so far and are often characterized by mild flu-like symptoms [2]. Public health issues associated with H9N2 viruses also include their ability to contribute genetic diversity to zoonotic avian influenza viruses (AIVs) with serious outcomes in humans. The internal gene segments of the AIVs responsible for fatal infections in humans (e.g. H5N1, H7N9, and more recently H5N6 and H10N8) are derived from H9N2 viruses [4–6].

Phylogenetically, 4 main H9 lineages have been reported in poultry: G1, Y280, Korean-like, and European lineages [7]. Understanding H9N2 circulation is critical for animal and human health, especially in Africa, where AIV circulation has intensified. Since 2006, the African continent has been affected by 3 waves of highly pathogenic (HP) H5 outbreaks. The first wave occurred in 2006 with H5N1 viruses from clade 2.2. The second wave occurred in 2015 and involved H5N1 viruses from clade 2.3.2.1c [8,9]. In 2016, clade 2.3.4.4B H5N8 viruses emerged in Western Africa and spread to Southern, Central, and Eastern Africa [9]. Although HP subtypes have been reported in Sub-Saharan Africa, sustained circulation of low pathogenic subtypes were not reported before 2017, despite active surveillance.

Surveillance studies before 2016 in domestic poultry in Western and Eastern Africa did not detect any LPAIV. Within the framework of active surveillance for animal influenza viruses we collected 26,746 swabs and 2276 sera from domestic poultry in Côte d’Ivoire, Benin, and Togo from 2008 through 2010 [10], and more were collected in the following years (unpublished data). None of those samples tested positive for AIVs, irrespective of subtype. A similar study conducted in Uganda in 2010–2011 showed a very low AIV positivity (1%) [11]. Since the emergence of G1 lineage H9N2 viruses in Morocco in early 2016, H9N2 viruses have been widely detected in Northern Africa, with spread to Western Africa, suggesting that the circulation of AIVs is markedly changing in the region. LPAI H9N2 viruses have continued to circulate in Morocco and Algeria [12].

Since 2017, H9N2 viruses have been detected in several Sub-Saharan African countries: Ghana, Burkina Faso, Uganda, Kenya, and Senegal, where a human case was recently reported [13–15]. A better understanding of the circulation and evolution of these viruses, is critical for animal and human health in Sub-Saharan Africa. We performed molecular and antigenic characterization, as well as growth kinetics for viruses from Benin, Togo, and Uganda to understand their genetic and antigenic evolution and replication pattern. We also assessed H9N2 antigenic drift, coupled with its putative effects on human and animal health, and the acquisition of mammalian adaptation markers.

## Materials and methods

### Sample collection, viral isolation, and sequencing

Through our surveillance programme implemented in Togo, Benin, and Uganda for influenza viruses in poultry, 4522 samples were collected from apparently healthy domestic birds in live birds markets (LBMs) and slaughterhouses between 2017 and 2019. Depending on the countries and the sampling sites, 10 to 461 samples were collected per site and per year. All samples from Benin and Uganda were collected from chickens. In Togo, most of the samples were from chickens (only 54 and 70 samples from guinea fowls and ducks, respectively). Collected oropharyngeal and cloacal swabs were pooled by mixing 5 samples. We purified total RNA with the QIAamp Viral RNA Mini kit (Qiagen). All pooled samples were tested by RT-PCR for the inﬂuenza A matrix gene [16]. When a pool was found positive, its composing samples were individually retested for the matrix gene. The positive individual swabs were then tested for the H9 gene [17] by using the iTaq Universal Syber Green One-Step kit (Bio-Rad). Viruses were isolated from positive samples in 10-day-old specific pathogen-free embryonated chicken eggs, followed by antigenic characterization and RNA extraction. Whole-genome sequencing of 3 isolates from Togo, 22 isolates from Benin, and 23 isolates from Uganda was performed with an Illumina MiSeq system, as previously described (GenBank accession nos. MW159147-MW159298, MW159300-MW159307, MW159309-MW159340, MW165126-MW165133) [18]. The libraries were generated by using an Illumina Nextera XT library prep kit (FC-131-1096). A tape station was used to verify the library quantity and quality.

### Molecular characterization

Consensus sequences of each gene segment of the AIV isolates were aligned with ClustalW, available in BioEdit, and BLAST (https://blast.ncbi.nlm.nih.gov/Blast.cgi?PROGRAM=blastn&PAGE_TYPE=BlastSearch&LINK_LOC=blasthome) searches of the Uganda and Benin sequences. The first 100 most-related sequences were selected, and the most closely related strains were selected for phylogenetic analysis. Phylogenetic trees were performed by using MegaX according to the general time reversible (GTR) model with a discrete gamma distribution (5 categories [+G]), performing a 1000 bootstrap resampling analysis. Phylogenetic trees were visualized by using FigTree, version 1.4.2 (https://tree.bio.ed.ac.uk/software/figtree). For the HA segments, phylogeny trees were generated with RaxML, version 8.2.X (https://sco.h-its.org/exelixis/software.html), with a GTR associated with gamma substitution model and then annotated with amino acid (aa) substitutions by using treesub (https://github.com/tamuri/treesub/blob/master/README.md). Phylogeny was visualized by using FigTree, version 1.4.2. The genetic similarity percentage was calculated by using the maximum composite likelihood model implemented in MegaX. We performed aa sequence alignments of all of the viral genes to identify substitutions previously associated with mammalian adaptation. Only the markers known to elicit biological effects *in vitro* or *in vivo* were considered. For the HA gene, only markers described for the H9N2 subtype were selected. The closest human strain was also considered for comparisons.

To identify any reassortment events among the Ugandan viruses on the basis of tree topology, genetic distances between groups were calculated with the maximum composite likelihood model. We defined the groups according to tree topology and intergroup genetic distances of >1% for each gene segment.

### Molecular clock analyses

The time to the most recent common ancestor (TMRCA) of the LPAI H9N2 viruses in Western Africa and Uganda were determined for the HA gene segment with BEAST, v1.7.1, software [19] implemented on a Galaxy workbench (https://vm-galaxy-prod.toulouse.inra.fr). The Hasegawa–Kishino–Yano + Gamma (4 categories) nucleotide substitution model was then applied with consideration of 2 codon positions (1st + 2nd or 3rd position) and an unlinked base frequency across all codon positions. A relaxed (uncorrelated lognormal) molecular clock and constant population size coalescent were specified as the tree priors. Markov chain Monte Carlo modelling with 100 × 10^6^ iterations was run and assessed with Tracer, v1.6 [20]. The maximum clade credibility phylogenetic tree with the mean TMRCA and their 95% highest posterior density (HPDs) were generated with TreeAnnotator, v1.8.1 [19], after a burn-in of 10% was applied. The tree was visualized with FigTree, v1.4.2.

### Antigenic characterization

Four isolates from Benin, 2 from Togo, and 1 from Uganda were selected based on phylogenetic analyses for antigenic characterization. Hemagglutination inhibition (HI) assays were performed as previously described [21] by using 8 polyclonal reference sera (7 from the G1 lineage, including 1 antiserum from Uganda, and 1 from the Y280 lineage; Appendix Table 1, see supplemental data). An antigenic map in 2 dimensions was made with Racmac software (https://github.com/acorg/Racmacs) implemented in Rstudio [22]. A column basis titre of 5120 was selected because it best represented the HI results according to the diagnosis plots.

**Table 1.** Number of samples collected per species and positivity rates over the surveillance period 2017-2019, in Togo, Benin, and Uganda.

	2017	2018	2019
Togo	**Nb. Swabs analysed (% A/H9N2 positive):** Chicken: 191 (4.2%) Guinea fowl: 16 (0%) Duck: 18 (5.6%) **Nb. Sampling sites:** 3 **Range % A/H9N2 positive/site:** [0–10.8]	**Nb. Swabs analysed (% A/H9N2 positive):** Chicken: 336 (0%) Guinea fowl: 21 (0%) Duck: 18 (0%) **Nb. Sampling sites:** 4	**Nb. Swabs analysed (% H9N2 positive):** Chicken: 464 (2.7%) Guinea fowl: 17 (5.8%) Duck: 34 (0%) **Nb. Sampling sites:** 12 **Range % A/H9N2 positive/site:** [0–12]
Benin	**Nb. Swabs analysed (% A/H9N2 positive):** Chicken: 120 (0%) **Nb. Sampling sites:** 2	**Nb. Swabs analysed (% A/H9N2 positive):** Chicken: 170 (6.5%) **Nb. Sampling sites:** 3 **Range % A/H9N2 positive/site:** [0–12.2]	**Nb. Swabs analysed (% A/H9N2 positive):** Chicken: 455 (24.2%) **Nb. Sampling sites:** 2 **Range % A/H9N2 positive/site:** [6.12–14.1]
Uganda	**Nb. Swabs analysed (% A/H9N2 positive):** Chicken: 692 (47%) **Nb. Sampling sites:** 4 **Range % A/H9N2 positive/site:** [41–66]	**Nb. Swabs analysed (% A/H9N2 positive):** Chicken: 967 (40%) **Nb. Sampling sites:** 4 **Range % A/H9N2 positive/site:** [32–53]	**Nb. Swabs analysed (% A/H9N2 positive):** Chicken: 948 (61%) **Nb. Sampling sites:** 4 **Range % A/H9N2 positive/site:** [49–68]

### Cell culture

MDCK cells and Calu-3 cells (ATCC) were cultured in modified Eagle’s medium (MEM; CellGro) containing 10% fetal calf serum (HyClone), 2 mM L-glutamine (Gibco), and 1x penicillin/streptomycin/amphotericin B (Gibco). Calu-3 cells were further supplemented with 1 mM sodium pyruvate (Gibco). Maintenance and viral infections were performed at 37°C, 5.5% CO_2_. Chicken red blood cells were purchased from Rockland Immunochemicals.

### Virus replication kinetics

Isolates selected for antigenic analysis were also characterized *in vitro* by growth kinetics in cultured human airway epithelial (Calu-3) cells. One isolate from Uganda with the mammalian adaptation mutation T190 V in the HA protein was also included in this experiment as well as two human A/ H9N2 strains as control (A/Hong Kong/1073/99 and A/Bangladesh/0994/2011) (Appendix Table 2, see supplemental data). Multi-step replication curves were performed in confluent calu-3 cells (5.4 × 10^5^ cells per well in 6 well plates). Monolayers were washed 2x with PBS and inoculated with 1 mL of the indicated virus (MOI 0.01) for 1 h at 37°C. Virus inoculums were removed, cells were washed with 0.9% saline (pH 2.0) to inactivate non-internalized virus particles, and washed an additional 2× with PBS (pH 7.2). Three mL of infection medium (MEM, 1% bovine serum albumin, 0.3 µg/mL TPCK trypsin, 1× penicillin/streptomycin/amphotericin B, 2 mM L-glutamine, 1 mM sodium pyruvate) was added to each well. Supernatants were collected between 2 and 96 h post-virus inoculation (hpi) and titered on MDCK cells in triplicate serial log_10_ dilution series per individual sample. Endpoint virus containing dilutions were determined by hemagglutination of 0.5% solutions of chicken red blood cells. Fifty percent tissue culture infectious doses were determined by method of Reed and Muench as described previously [23]. Two independent experiments were conducted, with 3 replicate wells per virus per experiment. Statistical analysis and graph for replication kinetics were performed using GraphPad Prism software. Statistical significance of replication between virus groups at a given time point was determined by performing a 2-way ANOVA. Results were considered statistically significant at *p*-value < 0.05.

## Results

### Surveillance data

Two sampling sites among 3 showed positive samples for LPAI H9N2 virus in 2017 in Togo. In 2018, all samples were collected in 4 new different sampling sites and no positive sample was found. In 2019, samples were collected in 12 different sampling sites including the sites already sampled in 2017 and in 2018. One sampling site found positive in 2017 was AIV positive again in 2019, one was negative in 2017 and became positive in 2019, and one site positive in 2017 was found negative in 2019. Two sampling sites negative in 2018 were found positive in 2019. The interpretation of results by species was difficult because of the many more chickens than ducks or guinea fowls sampled (Table 1).

In Benin, all samples were collected in chickens. In 2017 no positive sample was found. Later on, samples were found positive in 2018 and 2019. An additional sampling site was found positive in 2019 compared to 2018 in Benin (Table 1).

In Uganda, samples were collected in chickens, in the same four LBMs between 2017 and 2019, and showed very high positivity rates ranging between 32% and 68% (Table 1).

### Phylogenetic analysis

Phylogeny performed on the HA gene segment showed that all of the isolates from Benin, Togo, and Uganda belonged to the G1 lineage. The viruses from Benin shared 99.9% to 100% genetic identity, whereas the viruses from Togo and Uganda displayed higher genetic diversity. The intra-country diversity of the viruses in Togo and Uganda ranged from 0.2% to 0.4% and 0% to 1.3%, respectively.

**Figure 1.**
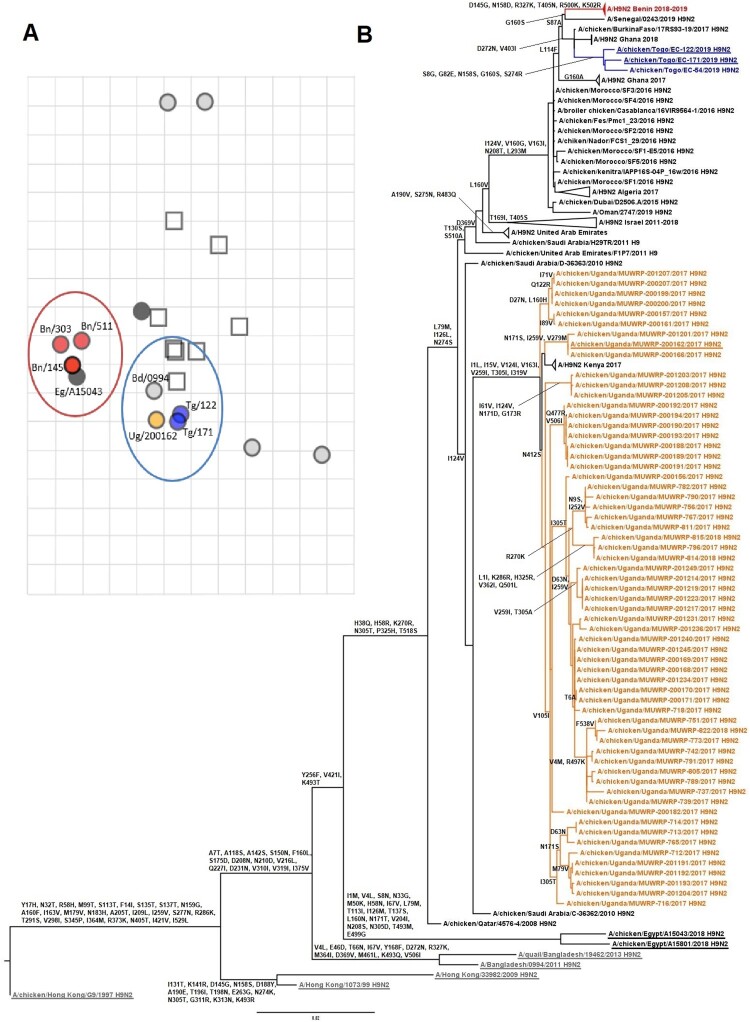
Antigenic and phylogenetic characterization of Sub-Saharan African H9N2 viruses. (A) Antigenic map resulting from HI assays of H9N2 viruses. The viruses are represented by circles and antisera by squares. One grid corresponds to 1 unit of antigenic distance, a 2-fold dilution in HI titres. (B) HA phylogenetic maximum-likelihood tree with synonymous substitutions. Scale bar indicates the number of nucleotide substitutions per site. Underlines depict the AIV H9N2 viruses used to draw the antigenic map. Influenza viruses from Togo are labelled in blue, Benin in red, Uganda in orange, Asia in grey, and Egypt underlined in black. (A/chicken/Uganda/MUWRP-790/2017: Ug/790; A/chicken/Uganda/MUWRP-200162/2017: Ug/200162; A/chicken/Togo/EC-171/2019: Tg/171; A/chicken/Togo/EC-122/2019: Tg/122; A/chicken/Benin/19-A-01-145-E/2019: Bn/145; A/chicken/Benin/19-A-02-303-E/2019: Bn/303; A/chicken/Benin/19-A-04-511-E/2019: Bn/511; A/Bangladesh/0994/2011: Bd/0994; A/chicken/Egypt/A15043/2018: Eg/A15043)

The isolates from Benin and Togo were closely related to the viruses previously identified in Western Africa. Both groups of viruses (Togo and Benin) displayed the highest genetic similarities with A/chicken/Burkina Faso/17RS93-19/2017 (99.6% genetic identity for viruses from Benin and 99.6%–99.7% for viruses from Togo) and 2018 Ghanaian viruses (genetic identity of 99.5% with viruses from Benin and 99.5%–99.6% for viruses from Togo). They all clustered together with recent Moroccan and Algerian H9N2 viruses (Figure 1). The viruses from Uganda clustered with H9N2 viruses from the Middle East collected in 2010 (97.7% genetic identity with the closest relative strain, A/chicken/Saudi Arabia/C-36362/2010), suggesting a distinct introduction in the region unrelated to the Northern African origin of the Western African strains. The long tree branch between viruses from Uganda and the Middle East, however, suggested a gap in surveillance and precluded determination of the origin of the H9N2 viruses isolated in Uganda (Figure 1A).

Whole-genome phylogeny supported the results observed for the HA segment phylogenetic tree, except for the strains from Uganda, where reassortment events between the different isolates were suspected (Appendix Figures 1–8, see supplemental data). We identified 6 groups of Ugandan viruses that were genetically distant by more than 1% on the basis of their tree topologies (Appendix Figure 8, see supplemental data). For the PB2 and PA genes, only 2 groups were identified, most likely because of the slower rate of substitutions in these genes, making the identification of possible reassortants unreliable. For the other genes, the different constellations of groups observed between the viruses for the different genes suggested reassortment events among strains from Uganda.

**Figure 2.**
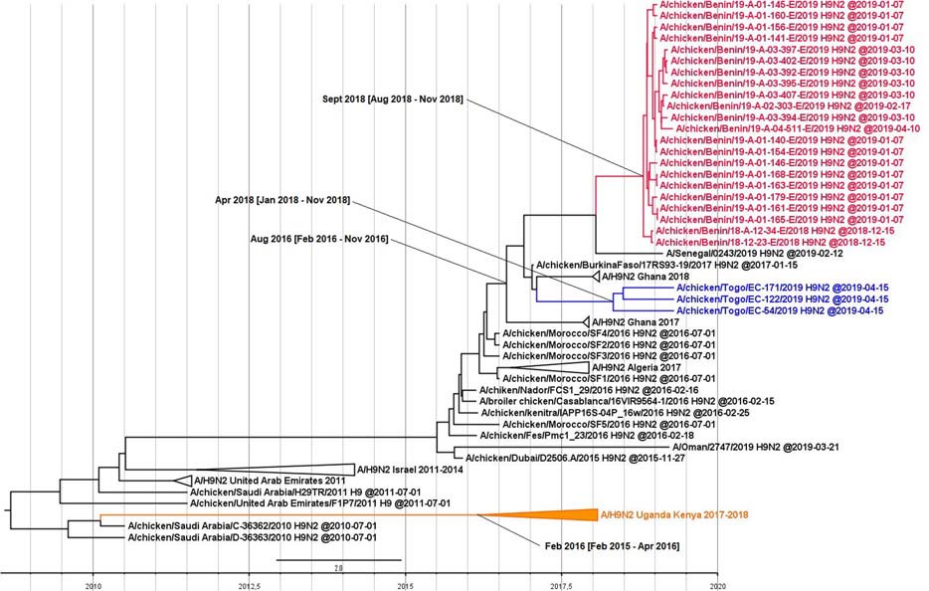
Time to the most recent common ancestor of Sub-Saharan African H9N2 viruses. Maximum clade credibility phylogenetic tree of the HA gene. The H9N2 viruses from Togo are represented in blue, Benin in red, and Uganda in orange. The mean TMRCA and the 95% highest posterior density intervals of the relevant nodes are indicated in parentheses.

On the basis of the HA sequences, we estimated the TMRCA of the H9N2 viruses in Western Africa as August 2016 (95% HPD interval from February 2016 to November 2016). We estimated the TMRCAs of the viruses from Benin and Togo as September 2018 (95% HPD interval of August–November 2018) and April 2018 (95% HPD interval of January–November 2018), respectively. We estimated the TMRCA of the H9N2 isolates from Eastern Africa as February 2016 (95% HPD interval of February 2015–April 2016) (Figure 2). Taken together, LPAI H9N2 viruses have likely emerged in Sub-Saharan Africa in 2016, with at least 2 distinct introductions: an introduction from the Middle East to East Africa first, and probably a few months later an introduction from North to West Africa.

### Molecular characterization and Mammalian adaptation marker identification

We observed a conserved HA cleavage site, RSSR/GLF, in all of the isolated viruses except for the isolates from Benin and 1 from Uganda, for which KSSR/GLF was identified. The receptor binding site was homogenous for most of the isolates with the following features: (1) All of the viruses in our study harboured the amino acids YWTHALY (located respectively in positions 101, 153, 155, 183, 190, 194, and 195; H3 numbering) except for 2 isolates from Uganda, which acquired A190T or A190V substitutions. (2) The right edge of the binding pocket (positions 138–142, H3 numbering) was GTSKS, and the left edge was NGLIGR (positions 224–229), except for 1 isolate from Uganda that harboured a SGLIGR motif. All of the isolated H9N2 viruses harboured the mammalian adaptation markers I155T and Q226L (H3 numbering) in the HA gene, which promote preferential binding to human-like α2-6-linked sialic acid receptors [24–26]. A potential additional glycosylation site in position 271–273 (H3 numbering), already reported for viruses from Ghana collected in 2018, also occurred in all of the H9N2 viruses from Togo [14]. Mutations with previously reported biological effects in mammals occurred in 6 other segments of H9N2 isolates from Benin, Togo, and Uganda (Appendix Table 2, see supplemental data).

### Antigenic properties

Antigenic analysis showed that most of the H9N2 viruses circulating in Sub-Saharan Africa were antigenically homogenous and displayed cross-reactivity with A/Bangladesh/0994/11, from which a World Health Organization nominated vaccine seed virus has been produced. Nevertheless, antigenic variation occurred in the viruses from Benin, which appeared more distant on the antigenic map than did the viruses from Togo and Uganda. This suggests that the acquisition of 1 or several substitutions on the HA gene segment of these viruses may be responsible for the decreased cross-reactivity with the other African isolates (Figure 1A). To identify the possible substitutions responsible for these 2 antigenic clusters, we compared the aa compositions of the viruses from Benin and Togo (i.e. the viruses most genetically similar among the H9N2 viruses reported on the antigenic map). The isolates from Benin and Togo differed by 10 substitutions in HA: G8S, D145G, S158D, N272D, R274S, R327K, I403V, T405N, R500K, and K502R, with only 5 located in HA1 and 1 in the cleavage site. The strain A/chicken/Egypt/A15043/2018 clustered with the viruses from Benin on the antigenic map but was very genetically distant from the viruses isolated in Western Africa, reducing our ability to identify the synonymous substitutions responsible for this observation.

### Growth kinetics of viruses in mammalian cells

**Figure 3.**
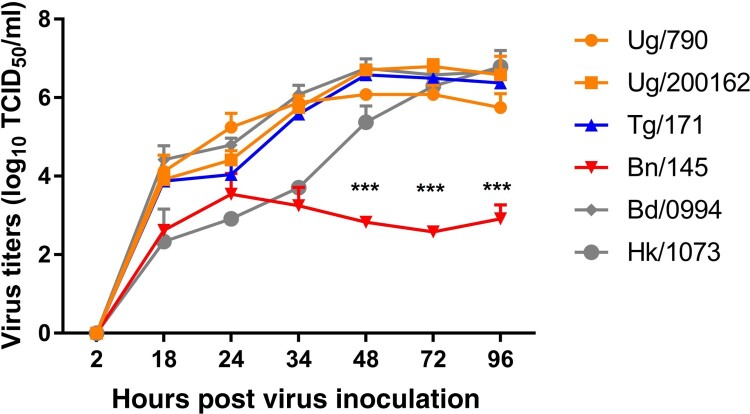
Replication kinetics of A/H9N2 viruses in Cultured Human Airway Epithelial Cells (Calu-3). Growth kinetics of Sub-Saharan Africa viruses were measured and compared with two A/H9N2 human strains after infection of Calu-3 cells with an MOI of 0.001, for indicated time points. Viruses from Uganda are labelled in orange, Benin in red, Togo in blue and the two Asian human strains in grey. Error bars indicate mean + SD of the combined results of 2 individual experiments performed in triplicate. Statistical significance of replication between virus groups at a given time point was determined by performing a 2-way ANOVA. *** *p*< 0.0001. (A/chicken/Uganda/MUWRP-790/2017: Ug/790; A/chicken/Uganda/MUWRP-200162/2017: Ug/200162; A/chicken/Togo/EC-171/2019: Tg/171; A/chicken/Benin/19-A-01-145-E/2019: Bn/145; A/Bangladesh/0994/2011: Bd/0994; A/Hong Kong/1073/99: Hk/1073).

Kinetics performed in Calu-3 cells showed a similar replication profile between viruses from Uganda and Togo and two reference Asian human strains. Nevertheless, the isolate from Benin, A/chicken/Benin/19-A-01-145-E/2019 showed significant lower virus titres compared to other viruses especially from 48 to 96 hpi (*p*-value < 0.0001) (Figure 3).

## Discussion

The spread of H9N2 viruses on the African continent has signalled a dramatic change in the ecology of AIV in the region. If these viruses follow the trends seen in other regions of the world, it is likely that they will become endemic. Surveillance carried out in Togo, Benin and Uganda between 2017 and 2019 showed an effective circulation of LPAI H9N2 viruses. While distinct locations were sampled in different months of different years in Benin and Togo, the number of positive sites globally increased with time. To investigate the impact of seasonality on the circulation of LPAI H9N2 viruses, samples should have been collected each month which unfortunately could not be done. Misdetection of H9N2 virus in Benin and Togo before 2017 is possible as very few sites were sampled. In contrast in Uganda, the same sites were sampled throughout the three years of surveillance with high positivity rates that ranged between 32% and 68%. The virus thus seems well established in these Ugandan LBMs, throughout the year, which was likely facilitated by the poor biosecurity.

Phylogenetic analyses revealed that all of the 48 studied LPAI H9N2 viruses belonged to the G1 lineage. The isolates from Benin and Togo clustered with viruses previously described in other Western Africa countries and LPAI H9N2 virus was very likely introduced in the region from Northern Africa, possibly through poultry trade, since the export of hatching eggs and day-old-chicks to West Africa from Morocco has been documented [27]. In addition, molecular clock analyses demonstrated that the TMRCA of the viruses from Western Africa was estimated as August 2016. The circulation of LPAI H9N2 viruses was not reported in Togo and Benin until 2017 despite animal influenza surveillance activities, suggesting a recent increase in H9N2 circulation in Sub-Saharan Africa.

Sequences analyses of the HA segment showed that isolates from Benin harboured a weak genetic diversity compared to viruses from Togo and Uganda, which can be explained by a recent introduction in Benin or by viruses’ origin, since viruses from Benin were collected in the same geographic area (i.e. the Parakou area). The isolates from Togo displayed higher genetic diversity than did the viruses from Benin, suggesting an earlier introduction in Togo than in Benin. This is supported by the TMRCA of the viruses from Togo of April 2018, 5 months earlier than the TMRCA of the viruses from Benin. For both groups of viruses, the 95% HPD intervals were large because of the limited number of available viral sequences from Western Africa. The differences of timing observed between the TMRCAs and the date of H9N2 first detection in several Western African countries, including Benin and Togo, show that H9N2 is difficult to detect in the field because it typically induces mild clinical signs in poultry.

The observed genetic diversity and continuous detection in 2017 and 2019 in Togo, since 2018 in Benin and along the three years of surveillance in Uganda suggest that circulation of H9N2 in Sub-Saharan Africa is approaching endemicity. The length of the tree branches for the isolates from Benin and Togo suggests that a gap in surveillance occurred, making a precise description of the viral circulation in the region difficult. The tree topology and difference in the genetic identity of the isolates from Uganda with their closest relative sequences also suggest a significant gap in surveillance in the Middle East and in Eastern Africa, rendering identification of the exact origin of the isolates from Uganda and Kenya impossible.

Antigenically, we identified 2 clusters, 1 comprising isolates from Togo and Uganda and the other isolates from Benin. Viruses from Togo and Uganda, despite diverging at the genetic level, displayed better cross-reactivity with the sera panel than did the viruses from Benin. The viruses from Benin and Togo, relatively similar to each other at the genetic level, differed by only 10 substitutions in the HA protein. Although performing reverse genetics studies is the only way to definitively identify the substitution or combination of substitutions responsible for this antigenic variation, position 145 harbouring a different aa composition between the isolates from Togo and Benin may account for their antigenic difference. Indeed, position 145 on the HA protein is known to play a role in antigenic variation and receptor binding in different subtypes. In H3N2 swine influenza viruses, position 145 demonstrates plasticity in aa composition without affecting viral replication *in vitro* but altering antigenicity [28]. More specifically, the D/G switch in position 145 (135 in H9 numbering) in H9N2 subtypes is not only responsible for modified antibody binding but also changes in receptor binding avidity, both described as immune escape mechanisms [29]. Some of the HA sequences from Benin differed in nucleotide composition without inducing any modifications in protein sequence. Nevertheless, the isolates from Benin were slightly distant from each other on the antigenic map, which may have resulted from viral quasispecies that were not detected by sequencing. Antigenic diversity is potentially more important than genetic diversity, especially regarding viruses form Uganda. One isolate was used for antigenic analysis despite the presence of marked genetic diversity among these viruses.

At least 59 human cases of H9N2 have been reported in the world [2]. In Africa, only 5 human cases have been reported since 2015, 4 in Egypt and 1 in Senegal. H9N2 viruses collected in poultry in Togo and Benin and the human strain A/Senegal/0243/2019 harboured the same profile of mammalian adaptation markers. Replication kinetics performed in human cells showed similar replication profiles for most of the African viruses with the Asian human strains used as controls. Nevertheless, the virus from Benin (Bn/145) showed a decrease of replicative fitness in human cells compared to other African and Asian viruses tested. *In vivo* characterizations were not performed since closely related viruses from Algeria and Ghana, and the human strain A/Hong Kong/1073/99 had already been characterized in chickens or ferrets [12,14,30]. Algerian viruses did not show any increase of virulence or airborne transmission ability in ferrets [12]. Similar results were observed in chicken with viruses from Ghana [14]. The human strain A/Hong Kong/1073/99 used in kinetics replication as control was also characterized in ferrets and did not show any increased virulence or airborne transmission ability [30]. These results suggest a similar zoonotic potential of Sub-Saharan African viruses with H9N2 isolated in human, except for the viruses from Benin which showed an antigenic dritft and a decrease of replication in human cells. Putative human cases may have been missed in the region because the H9N2 virus is generally associated with mild disease [1]. Vaccinating poultry against LPAI A/H9N2 viruses, as occurs in Northern Africa, may decrease viral load and therefore prevent transmission of H9N2 viruses from poultry to humans. However, it may also add a selective pressure on the viruses and lead to an increased substitution rate and faster phenotypic evolution [31].

In conclusion, the phylogenetic analysis of the H9N2 isolates collected in poultry in Togo, Benin, and Uganda between 2017 and 2019 supports the trend of LPAI H9N2 viruses becoming endemic in several regions in Africa. Phylogenetic analysis, sequence comparison with the human strain isolated in Senegal in 2019 and kinetics replication profiles in human cells suggest that these viruses might have the ability to infect humans. Surveillance of the zoonotic strains currently spreading in the region must be continued to protect animal health and prevent more human cases or the emergence of a new putative zoonotic subtypes. The circulation of HPAI H5N8 and H5N6 recently reported in neighbouring countries increases the threat of emergence of new potential zoonotic viruses by reassortment with LPAI H9N2 in Sub-Saharan Africa [9,32]. Our antigenic analysis revealed the occurrence of antigenic drift. Since poultry vaccination campaigns against H9N2 viruses have started in Northern African, a wider drift can be expected in the future. Further studies, including those of vaccine-induced selective pressure on H9N2 evolution in Africa, are warranted to prevent both further economic losses for the poultry industry and public health threats.

## Supplementary Material

Appendix_Table_2.docx

Appendix_Table_1.docx

Appendix_Figure_Legends.docx

Supplemental_Figure_8.docx

Supplemental_Figure_7__NS_.JPEG

Supplemental_Figure_6__M_.JPEG

Supplemental_Figure_5__NA_.JPEG

Supplemental_Figure_4__NP_.JPEG

Supplemental_Figure_3__PA_.JPEG

Supplemental_Figure_2__PB1_.JPEG

Supplemental_Figure_1__PB2_.JPEG
