# Supplementary material for: Antigenic and molecular characterization of low pathogenic avian influenza A(H9N2) viruses in sub-Saharan Africa from 2017 through 2019
Source: Emerg Microbes Infect. 2021 Mar 23;10(1):753–61. doi: 10.1080/22221751.2021.1908097 (PMC8057090; doi:10.1080/22221751.2021.1908097)
Supplement: Appendix_Table_2.docx [file TEMI_A_1908097_SM8057.docx]

**Appendix Table 2.** Experimentally verified mammalian molecular markers of H9N2 isolates in Benin, Togo, Uganda, and Senegal

| Gene | Substitution | Benin | Togo | Uganda | Senegal | Phenotype | Reference |
| --- | --- | --- | --- | --- | --- | --- | --- |
| PB2 | D9N | D | D | D/N | D | Increased virulence in mice (H5N1) | [1,2] |
|  | K526R | K | K | K/R | K | Increased polymerase activity in mammalian cell line (H5N1) | [3] |
|  | T588I | A | A | I | A | Increased polymerase activity in mammalian cell line | [4] |
|  | V598T/I | T | T | T | T | Increased polymerase activity and replication in mammalian cells, increased virulence in mice (H7N9) | [5] |
|  | L89V, G309D | V  D | L/V  D | V  D | V  D | Increased polymerase activity in mammalian cell line and increased virulence in mice (H5N1) | [6] |
|  | L89V  G309D  T339K  R477G  I495V  A676T | V  D  K  G  V  I | L/V  D  K  G  V  V | V  D  K  G  I  V | V  D  K  G  V  V | Increased polymerase activity in mammalian cell line and increased virulence in mice, compensate PB2 E627K (H5N1) | [6] |
| PB1 | D3V | V | V | V | – | Increased polymerase activity and viral replication in avian and mammalian cell lines (H5N1) | [7] |
|  | K207R | K | K | K | K | Decreased polymerase activity in mammalian cells (H5N1) | [8] |
|  | D622G | G | G | G | G | Increased polymerase activity and virulence in mice (H5N1) | [9] |
| PA | S37A | A | A | A | A | Increased polymerase activity in mammalian cell lines (H7N9) | [10] |
|  | N383D | D | D | D | D | Increased polymerase activity in mammalian and avian cell lines (H5N1) | [11,12] |
| HA | T190V | A | A | A/T/V | A | Enhances binding affinity to mammalian cells and replication in mammalian cells; Involved in antibody binding variation | [13,14] |
|  | I155T | T | T | T | T | Increased virus binding to α2,6 | [15] |
|  | Q226L | L | L | L | L | Increased virus binding to α2–6, enhanced replication in mammalian cells and ferrets, enhanced contact transmission in ferrets | [16,17] |
| NP | M105V | V | V | V | V | Increased virulence in chicken (H5N1) | [18] |
|  | A184K | K | K | K | K | Increased virulence in chicken (H5N1) | [19] |
|  | K198R | K | K | K | K | Decreased polymerase activity in mammalian cells (H5N1) | [20] |
|  | E210D | D | D | D | D | Increased polymerase activity in mammalian cell line (H7N9) | [21] |
| MP (M1) | N30D | D | D | D | D | Increased virulence in mice (H5N1) | [22] |
|  | I43M | M | M | M | M | Increased virulence in mice, chickens, and ducks (H5N1) | [23] |
|  | T215A | A | A | A | A | Increased virulence in mice (H5N1) | [22] |
| MP (M2) | S31N | N | N | N | N | Increased resistance to amantadine | [24] |
| NS1 | P42S | S | S | S | S | Increased virulence and decreased antiviral response in mice (H5N1) | [25] |
|  | I106M | M | M | M | M | Increased viral replication in mammalian cells and virulence in mice (H1N1) with all internal genes from H7N9 | [26] |

1 Kim JH, Hatta M, Watanabe S *et al*. Role of host-specific amino acids in the pathogenicity of avian H5N1 influenza viruses in mice. *J Gen Virol* 2010; **91**: 1284–1289.

2 Graef KM, Vreede FT, Lau Y-F *et al.* The PB2 Subunit of the Influenza Virus RNA Polymerase Affects Virulence by Interacting with the Mitochondrial Antiviral Signaling Protein and Inhibiting Expression of Beta Interferon. *J Virol* 2010; **84**: 8433–8445.

3 Song W, Wang P, Mok BW-Y *et al.* The K526R substitution in viral protein PB2 enhances the effects of E627K on influenza virus replication. *Nat Commun* 2014; **5**: 5509.

4 Lee C-Y, An S-H, Choi J-G *et al*. Rank orders of mammalian pathogenicity-related PB2 mutations of avian influenza A viruses. *Sci Rep* 2020; **10**: 5359.

5 Hu M, Yuan S, Zhang K *et al.* PB2 substitutions V598T/I increase the virulence of H7N9 influenza A virus in mammals. *Virology* 2017; **501**: 92–101.

6 Li J, Ishaq M, Prudence M *et al.* Single mutation at the amino acid position 627 of PB2 that leads to increased virulence of an H5N1 avian influenza virus during adaptation in mice can be compensated by multiple mutations at other sites of PB2. *Virus Res* 2009; **144**: 123–129.

7 Elgendy EM, Arai Y, Kawashita N *et al.* Identification of polymerase gene mutations that affect viral replication in H5N1 influenza viruses isolated from pigeons. *J Gen Virol* 2017; **98**: 6–17.

8 Hulse-Post DJ, Franks J, Boyd K *et al.* Molecular Changes in the Polymerase Genes (PA and PB1) Associated with High Pathogenicity of H5N1 Influenza Virus in Mallard Ducks. *J Virol* 2007; **81**: 8515–8524.

9 Feng X, Wang Z, Shi J *et al.* Glycine at Position 622 in PB1 Contributes to the Virulence of H5N1 Avian Influenza Virus in Mice. *J Virol* 2016; **90**: 1872–1879.

10 Yamayoshi S, Yamada S, Fukuyama S *et al.* Virulence-Affecting Amino Acid Changes in the PA Protein of H7N9 Influenza A Viruses. *J Virol* 2014; **88**: 3127–3134.

11 Song J, Xu J, Shi J, Li Y, Chen H. Synergistic Effect of S224P and N383D Substitutions in the PA of H5N1 Avian Influenza Virus Contributes to Mammalian Adaptation. *Sci Rep* 2015; **5**.

12 Song J, Feng H, Xu J *et al.* The PA protein directly contributes to the virulence of H5N1 avian influenza viruses in domestic ducks. *J Virol* 2010; **85**: 2180–2188.

13 Teng Q, Xu D, Shen W *et al.* A Single Mutation at Position 190 in Hemagglutinin Enhances Binding Affinity for Human Type Sialic Acid Receptor and Replication of H9N2 Avian Influenza Virus in Mice. *J Virol* 2016; **90**: 9806–9825.

14 Yang W, Punyadarsaniya D, Lambertz RLO *et al.* Mutations during the Adaptation of H9N2 Avian Influenza Virus to the Respiratory Epithelium of Pigs Enhance Sialic Acid Binding Activity and Virulence in Mice. *J Virol* 2017; **91**.

15 Li X, Shi J, Guo J *et al.* Genetics, Receptor Binding Property, and Transmissibility in Mammals of Naturally Isolated H9N2 Avian Influenza Viruses. *PLoS Pathog* 2014; **10**.

16 Wan H, Perez DR. Amino acid 226 in the hemagglutinin of H9N2 influenza viruses determines cell tropism and replication in human airway epithelial cells. *J Virol* 2007; **81**: 5181–5191.

17 Wan H, Sorrell EM, Song H *et al.* Replication and transmission of H9N2 influenza viruses in ferrets: evaluation of pandemic potential. *PloS One* 2008; **3**: e2923.

18 Tada T, Suzuki K, Sakurai Y *et al.* NP Body Domain and PB2 Contribute to Increased Virulence of H5N1 Highly Pathogenic Avian Influenza Viruses in Chickens. *J Virol* 2011; **85**: 1834–1846.

19 Wasilenko JL, Sarmento L, Pantin-Jackwood MJ. A single substitution in amino acid 184 of the NP protein alters the replication and pathogenicity of H5N1 avian influenza viruses in chickens. *Arch Virol* 2009; **154**: 969–979.

20 Chen L, Wang C, Luo J *et al.* Amino Acid Substitution K470R in the Nucleoprotein Increases the Virulence of H5N1 Influenza A Virus in Mammals. *Front Microbiol* 2017; **8**.

21 Zhu W, Zou X, Zhou J *et al*. Residues 41V and/or 210D in the NP protein enhance polymerase activities and potential replication of novel influenza (H7N9) viruses at low temperature. *Virol J* 2015; **12**.

22 Fan S, Deng G, Song J *et al.* Two amino acid residues in the matrix protein M1 contribute to the virulence difference of H5N1 avian influenza viruses in mice. *Virology* 2009; **384**: 28–32.

23 Nao N, Kajihara M, Manzoor R *et al.* A Single Amino Acid in the M1 Protein Responsible for the Different Pathogenic Potentials of H5N1 Highly Pathogenic Avian Influenza Virus Strains. *PloS One* 2015; **10**: e0137989.

24 Ilyushina NA, Govorkova EA, Webster RG. Detection of amantadine-resistant variants among avian influenza viruses isolated in North America and Asia. *Virology* 2005; **341**: 102–106.

25 Jiao P, Tian G, Li Y *et al.* A Single-Amino-Acid Substitution in the NS1 Protein Changes the Pathogenicity of H5N1 Avian Influenza Viruses in Mice. *J Virol* 2008; **82**: 4190.

26 Ayllon J, Domingues P, Rajsbaum R *et al.* A single amino acid substitution in the novel H7N9 influenza A virus NS1 protein increases CPSF30 binding and virulence. *J Virol* 2014; **88**: 12146–12151.
