# Supplementary material for: Antigenic and molecular characterization of low pathogenic avian influenza A(H9N2) viruses in sub-Saharan Africa from 2017 through 2019
Source: Emerg Microbes Infect. 2021 Mar 23;10(1):753–61. doi: 10.1080/22221751.2021.1908097 (PMC8057090; doi:10.1080/22221751.2021.1908097)
Supplement: Appendix_Table_1.docx [file TEMI_A_1908097_SM8056.docx]

**Appendix Table 1.** Antigenic properties of selected Sub-Saharan H9N2 isolates, as measured by the hemagglutination inhibition assay

| H9N2 isolate | Clade | HK/G9 | Bd/0994 | HK/1073 | HK/33982 | Quail/Bd/2013 | Ug/200162 | Eg/15043 | Eg/15801 |
| --- | --- | --- | --- | --- | --- | --- | --- | --- | --- |
| A/chicken/Hong Kong/G9/97-PR8-IBCDC-2 | Y280 | **160** | 80 | 10 | 10 | 40 | 80 | 80 | 20 |
| A/Bangladesh/0994/2011-IDCDC-RG31 | G1 | 320 | **5120** | 160 | 160 | 640 | 1280 | 1280 | 640 |
| A/Hong Kong /1073/97 | G1 | 10 | 10 | **320** | 320 | 10 | 10 | 20 | 10 |
| A/Hong Kong/33982/2009-PR8-IDCDC-RG-26 | G1 | 20 | 10 | 80 | **1280** | 10 | 10 | 10 | 10 |
| A/quail/Bangladesh/19462/2013 | G1 | 160 | 320 | 40 | 20 | **640** | 160 | 320 | 80 |
| A/chicken/Uganda/200162/2017 | G1 | 160 | 2560 | 80 | 40 | 640 | **1280** | 640 | 320 |
| A/chicken/Egypt/A15043/2018 | G1 | 80 | 320 | 40 | 40 | 160 | 320 | **640** | 640 |
| A/chicken/Egypt/A15801/2018 | G1 | 320 | 640 | 320 | 640 | 640 | 1280 | 2560 | **5120** |
| A/chicken/Benin/18-A-12-23-E/2018 | G1 | 80 | 320 | 40 | 80 | 320 | 320 | 320 | 320 |
| A/chicken/Benin/19-A-01-145-E/2019 | G1 | 80 | 320 | 40 | 80 | 320 | 320 | 320 | 320 |
| A/chicken/Benin/19-A-02-303-E/2019 | G1 | 80 | 160 | 40 | 80 | 160 | 320 | 320 | 320 |
| A/chicken/Benin/19-A-04-511-E/2019 | G1 | 80 | 320 | 80 | 160 | 320 | 640 | 640 | 320 |
| A/chicken/Togo/EC-122/2019 | G1 | 320 | 2560 | 80 | 40 | 1280 | 1280 | 1280 | 320 |
| A/chicken/Togo/EC-171/2019 | G1 | 320 | 2560 | 80 | 40 | 640 | 640 | 1280 | 320 |

Numbers in bold font correspond to homologous HI titers.
