# Supplementary material for: Antigenic and molecular characterization of low pathogenic avian influenza A(H9N2) viruses in sub-Saharan Africa from 2017 through 2019
Source: Emerg Microbes Infect. 2021 Mar 23;10(1):753–61. doi: 10.1080/22221751.2021.1908097 (PMC8057090; doi:10.1080/22221751.2021.1908097)
Supplement: Appendix_Figure_Legends.docx [file TEMI_A_1908097_SM8055.docx]

**Appendix Figure Legends**

**Figure 1.** Maximum likelihood phylogenetic tree of the PB2 gene. The IAV H9N2 viruses from Togo are represented in blue, Benin in red, and Uganda in orange. Bootstrap supports >70% are indicated next to the nodes; scale bar indicates the numbers of nucleotide substitutions per site.

**Figure 2.** Maximum likelihood phylogenetic tree of the PB1 gene. The AIV H9N2 viruses from Togo are represented in blue, Benin in red, and Uganda in orange. Bootstrap supports >70% are indicated next to the nodes; scale bar indicates the numbers of nucleotide substitutions per site.

**Figure 3.** Maximum likelihood phylogenetic tree of the PA gene. The AIV H9N2 viruses from Togo are represented in blue, Benin in red, and Uganda in orange. Bootstrap supports >70% are indicated next to the nodes; scale bar indicates the numbers of nucleotide substitutions per site.

**Figure 4.** Maximum likelihood phylogenetic tree of the NP gene. The AIV H9N2 viruses from Togo are represented in blue, Benin in red, and Uganda in orange. Bootstrap supports >70% are indicated next to the nodes; scale bar indicates the numbers of nucleotide substitutions per site.

**Figure 5.** Maximum likelihood phylogenetic tree of the NA gene. The AIV H9N2 viruses from Togo are represented in blue, Benin in red, and Uganda in orange. Bootstrap supports >70% are indicated next to the nodes; scale bar indicates the numbers of nucleotide substitutions per site.

**Figure 6.** Maximum likelihood phylogenetic tree of the matrix protein gene. The AIV H9N2 viruses from Togo are represented in blue, Benin in red, and Uganda in orange. Bootstrap supports >70% are indicated next to the nodes; scale bar indicates the numbers of nucleotide substitutions per site.

**Figure 7.** Maximum likelihood phylogenetic tree of the NS gene. The AIV H9N2 viruses from Togo are represented in blue, Benin in red, and Uganda in orange. Bootstrap supports >70% are indicated next to the nodes; scale bar indicates the numbers of nucleotide substitutions per site.

**Figure 8.** Gene segment constellations of AIV H9N2 isolates from Uganda.

For each gene segment, the viruses (depicted in different colors) appeared distant according to the tree topology and displayed a genetic distance superior to 1%, which was calculated with the maximum likelihood model. Six groups were defined and named according to their oldest strain. When no difference was observed between 2 previously identified groups for a gene segment, the merged group was named according to the oldest strain.
