# Supplementary figures and images for: Antigenic and molecular characterization of low pathogenic avian influenza A(H9N2) viruses in sub-Saharan Africa from 2017 through 2019
Source: Emerg Microbes Infect. 2021 Mar 23;10(1):753–61. doi: 10.1080/22221751.2021.1908097 (PMC8057090; doi:10.1080/22221751.2021.1908097)

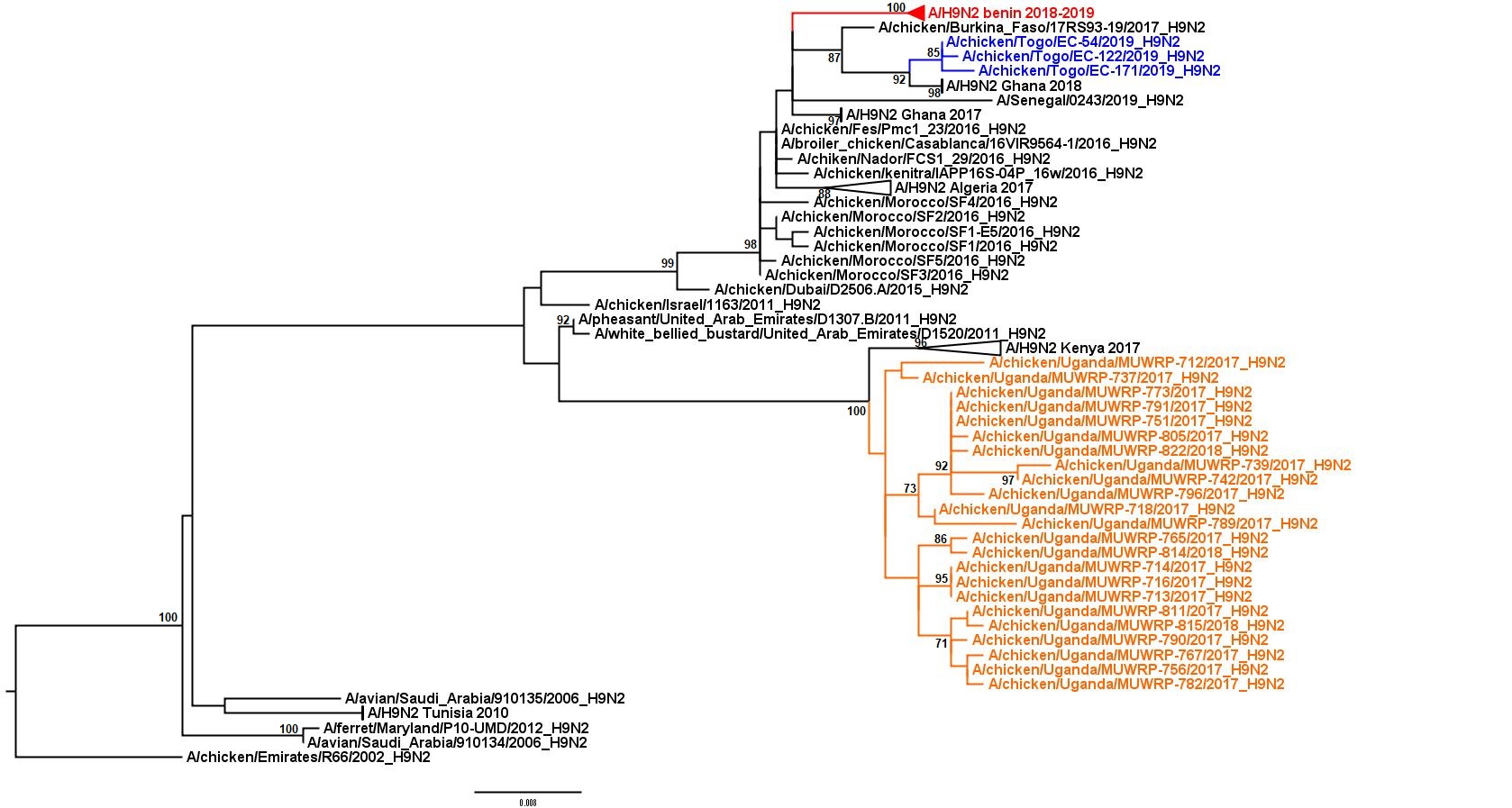

Supplement: Supplemental_Figure_7__NS_.JPEG [file TEMI_A_1908097_SM8053.jpeg]

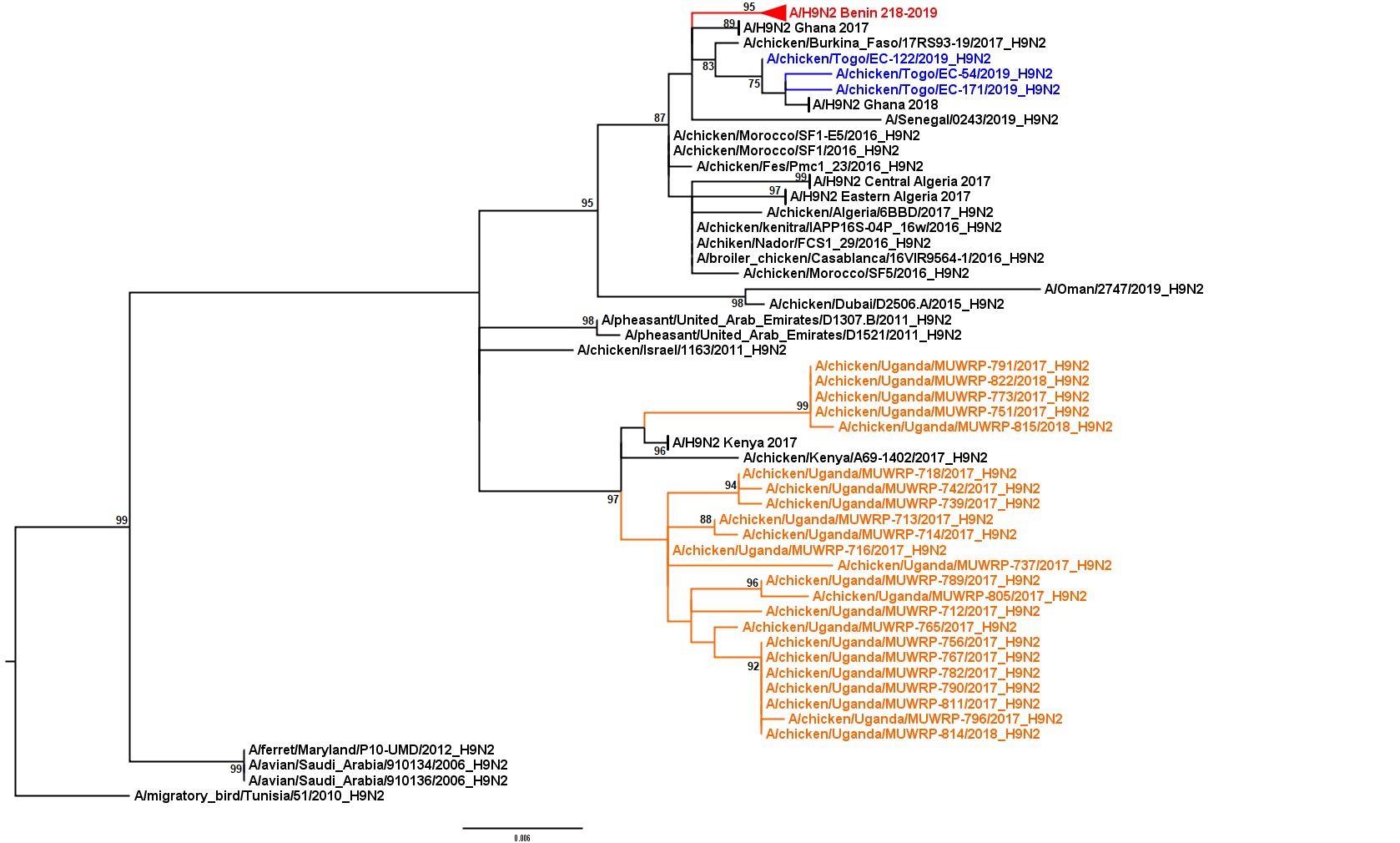

Supplement: Supplemental_Figure_6__M_.JPEG [file TEMI_A_1908097_SM8052.jpeg]

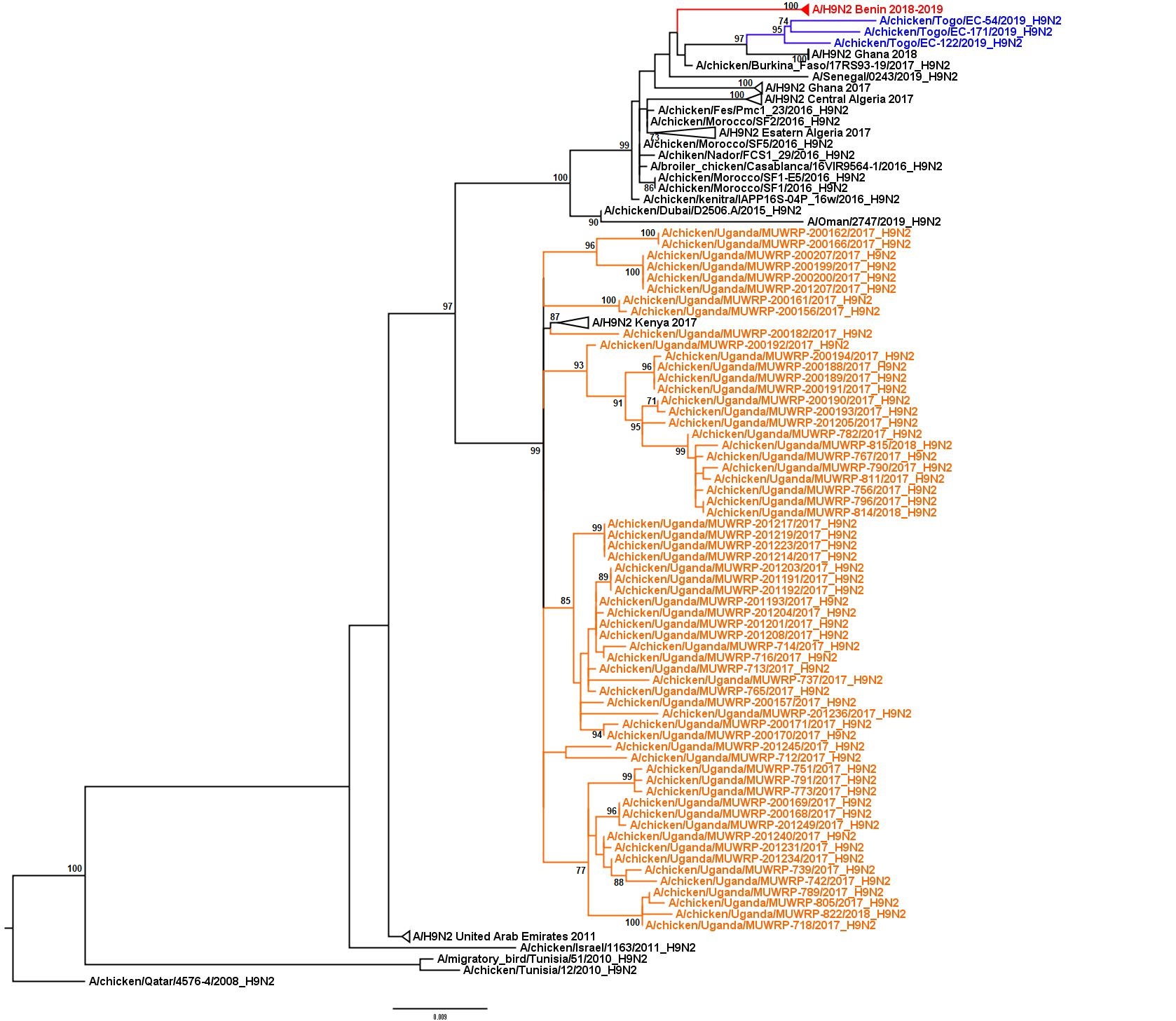

Supplement: Supplemental_Figure_5__NA_.JPEG [file TEMI_A_1908097_SM8051.jpeg]

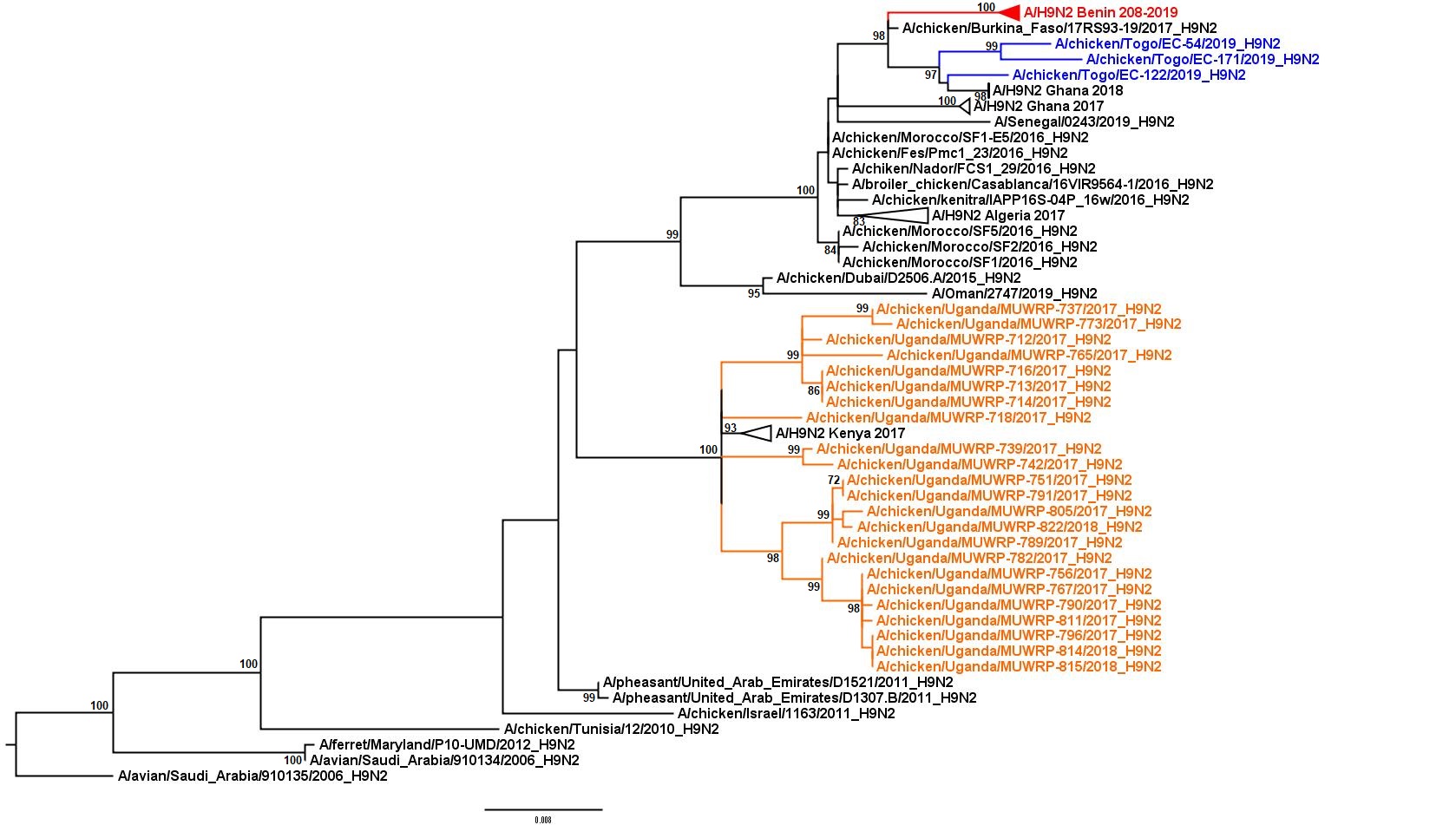

Supplement: Supplemental_Figure_4__NP_.JPEG [file TEMI_A_1908097_SM8050.jpeg]

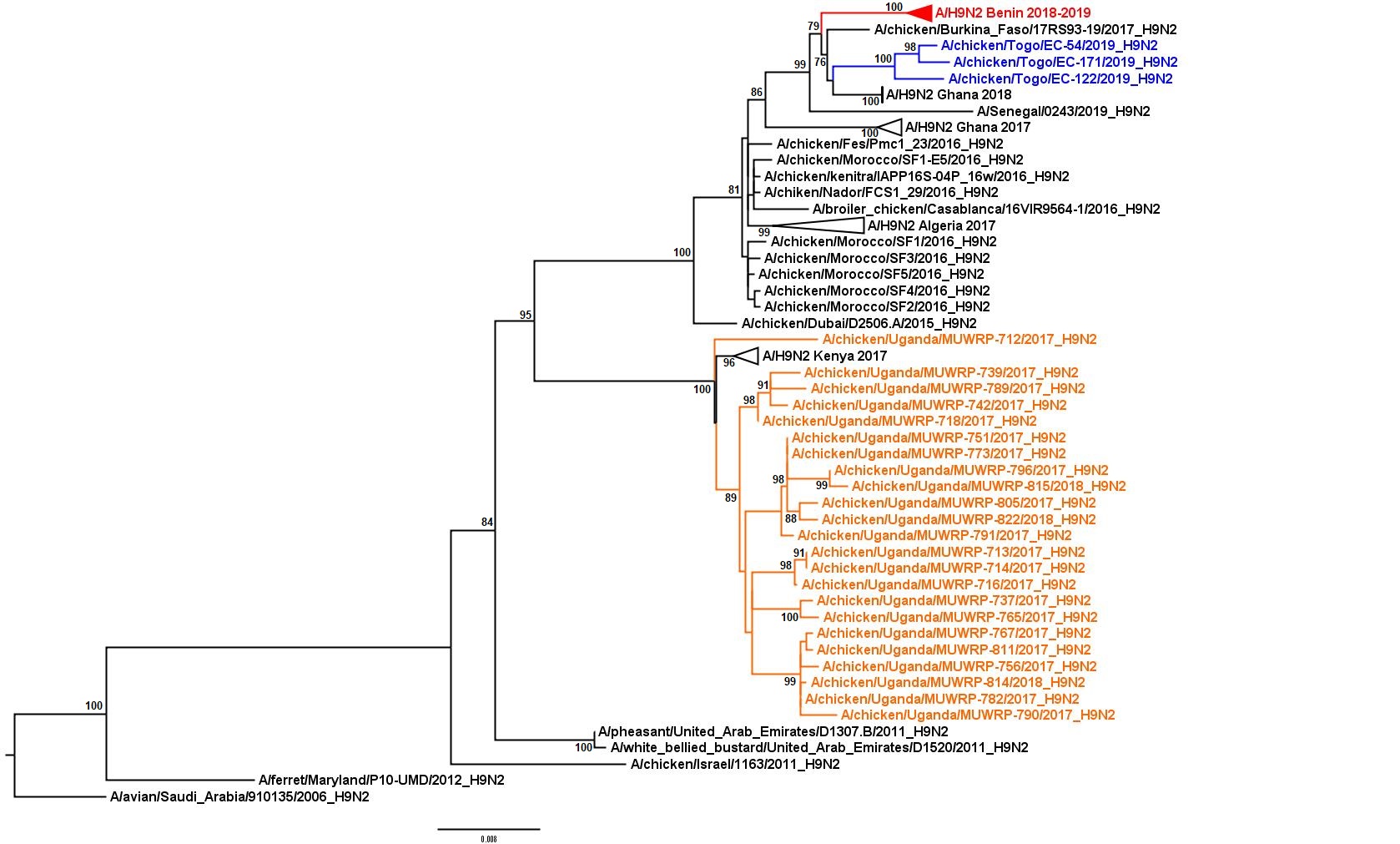

Supplement: Supplemental_Figure_3__PA_.JPEG [file TEMI_A_1908097_SM8049.jpeg]

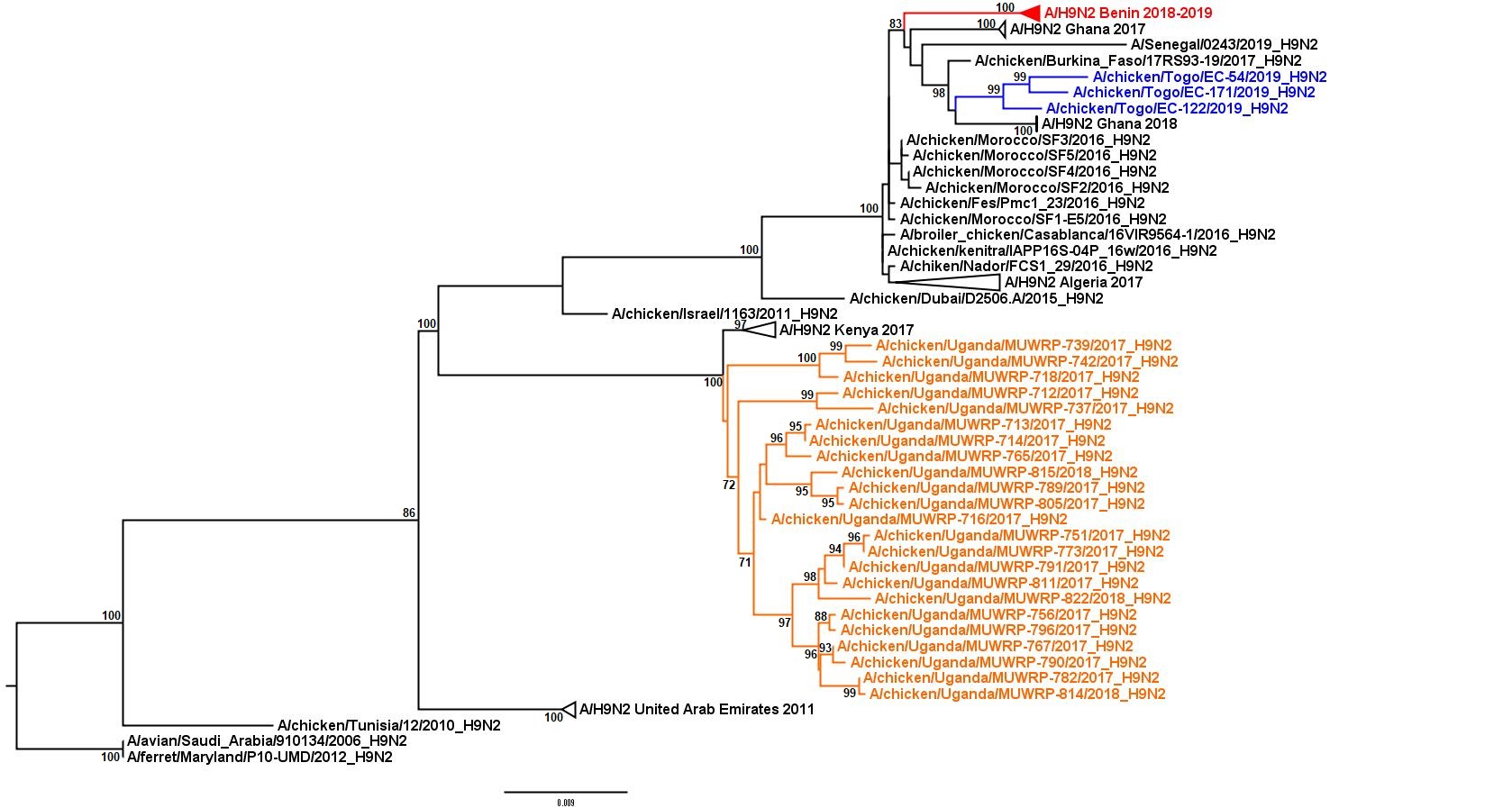

Supplement: Supplemental_Figure_2__PB1_.JPEG [file TEMI_A_1908097_SM8048.jpeg]

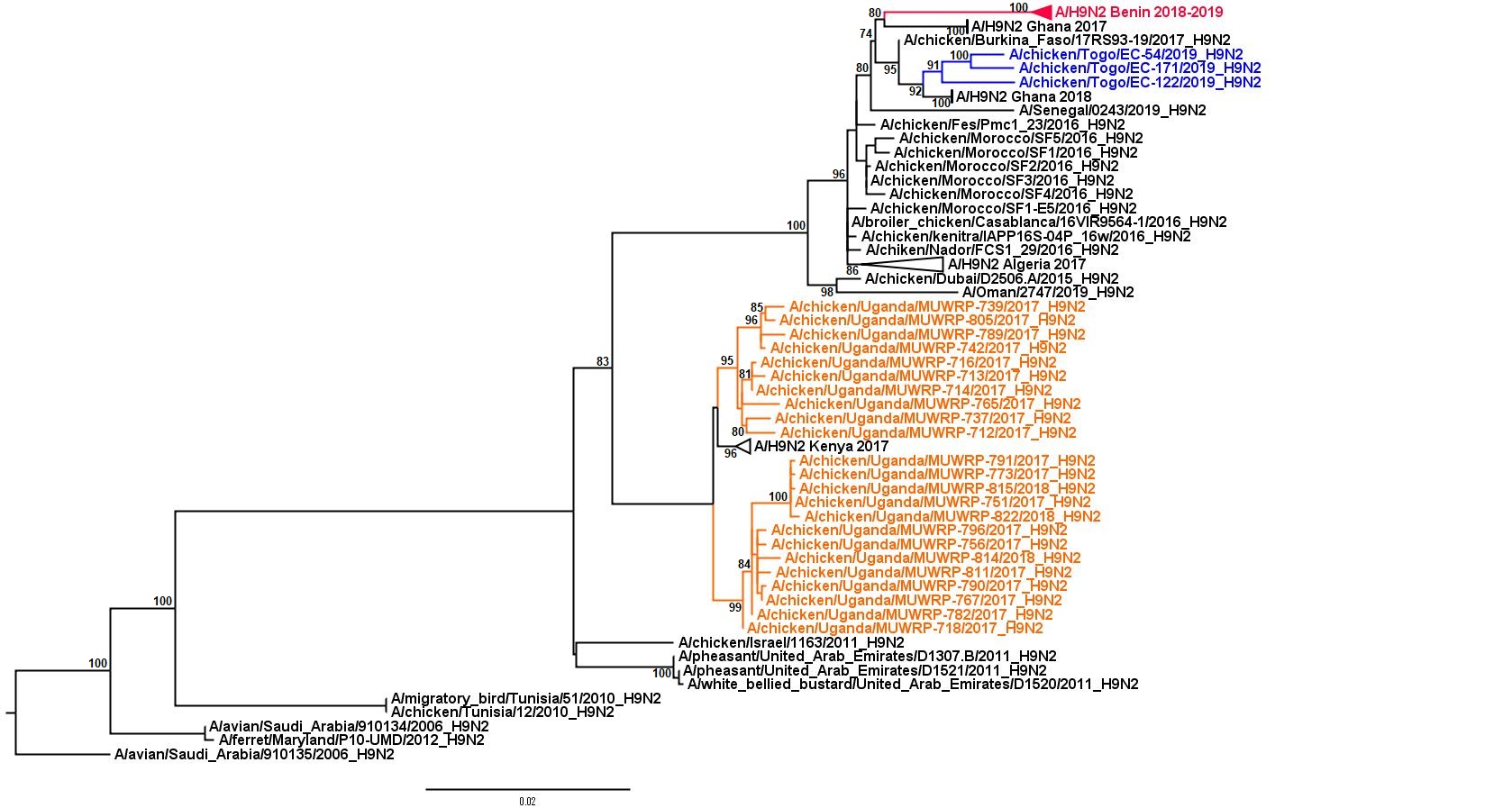

Supplement: Supplemental_Figure_1__PB2_.JPEG [file TEMI_A_1908097_SM8047.jpeg]
