# Supplementary material for: Antigenic and molecular characterization of low pathogenic avian influenza A(H9N2) viruses in sub-Saharan Africa from 2017 through 2019
Source: Emerg Microbes Infect. 2021 Mar 23;10(1):753–61. doi: 10.1080/22221751.2021.1908097 (PMC8057090; doi:10.1080/22221751.2021.1908097)
Supplement: Supplementary file 5 [file TEMI_A_1908097_SM8054.docx]

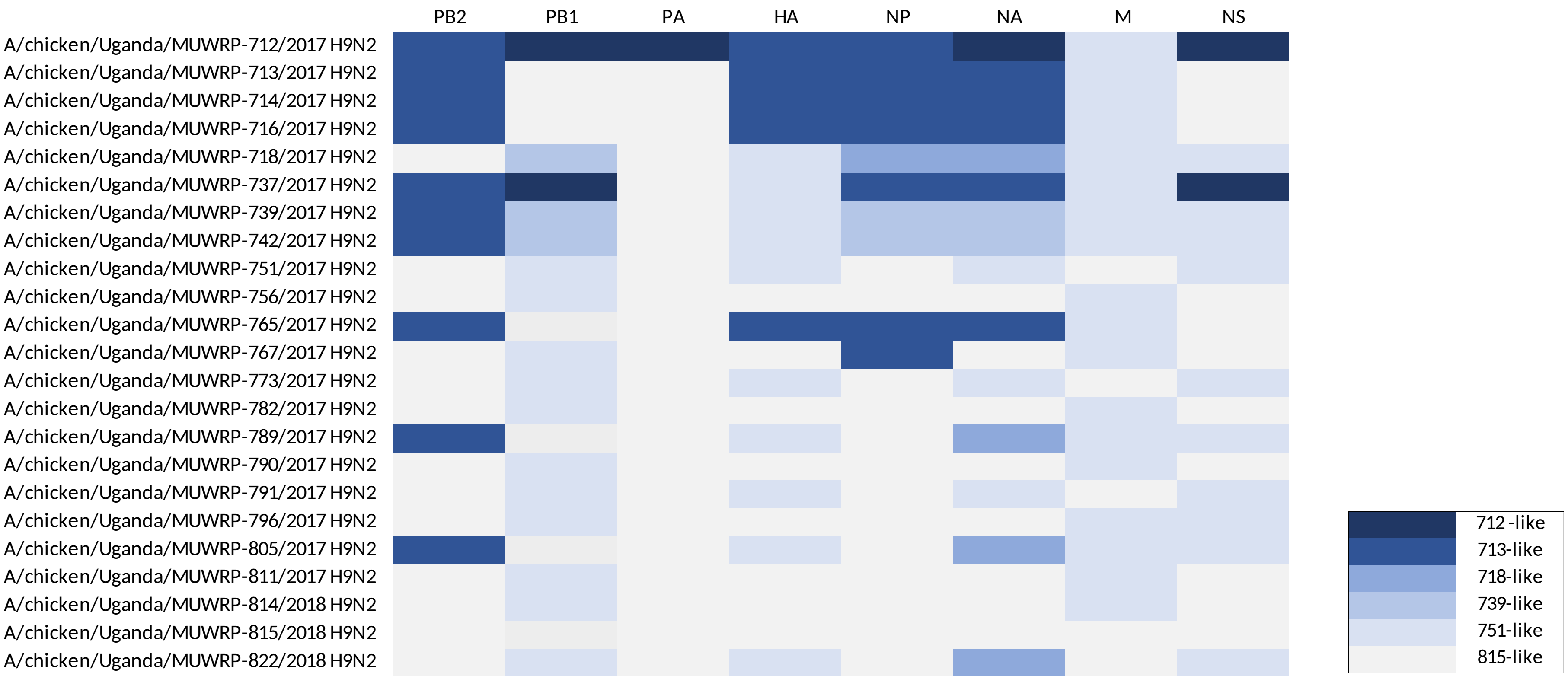
PB2
PB1
PA
HA
NP
NA
M
NS
A/chicken/Uganda/MUWRP-712/2017 H9N2
A/chicken/Uganda/MUWRP-713/2017 H9N2
A/chicken/Uganda/MUWRP-714/2017 H9N2
A/chicken/Uganda/MUWRP-716/2017 H9N2
A/chicken/Uganda/MUWRP-718/2017 H9N2
A/chicken/Uganda/MUWRP-737/2017 H9N2
A/chicken/Uganda/MUWRP-739/2017 H9N2
A/chicken/Uganda/MUWRP-742/2017 H9N2
A/chicken/Uganda/MUWRP-751/2017 H9N2
A/chicken/Uganda/MUWRP-756/2017 H9N2
A/chicken/Uganda/MUWRP-765/2017 H9N2
A/chicken/Uganda/MUWRP-767/2017 H9N2
A/chicken/Uganda/MUWRP-773/2017 H9N2
A/chicken/Uganda/MUWRP-782/2017 H9N2
A/chicken/Uganda/MUWRP-789/2017 H9N2
A/chicken/Uganda/MUWRP-790/2017 H9N2
A/chicken/Uganda/MUWRP-791/2017 H9N2
A/chicken/Uganda/MUWRP-796/2017 H9N2
A/chicken/Uganda/MUWRP-805/2017 H9N2
A/chicken/Uganda/MUWRP-811/2017 H9N2
A/chicken/Uganda/MUWRP-814/2018 H9N2
A/chicken/Uganda/MUWRP-815/2018 H9N2
A/chicken/Uganda/MUWRP-822/2018 H9N2
712 -like
713-like
718-like
739-like
751-like
815-like
